# Endoscopically assisted resection of a scapular osteochondroma causing snapping scapula syndrome

**DOI:** 10.1186/1477-7819-5-37

**Published:** 2007-03-22

**Authors:** Satoru Fukunaga, Hiroyuki Futani, Shinichi Yoshiya

**Affiliations:** 1 Department of Orthopedic Surgery, Hyogo Medical University, Hyogo, Japan

## Abstract

**Background:**

Osteochondroma is the most common benign bone tumor in the scapula. This condition might lead to snapping scapula syndrome, which is characterized by painful, audible, and/or palpable abnormal scapulothoracic motion. In the present case, this syndrome was successfully treated by use of endoscopically assisted resection of the osteochondroma.

**Case presentation:**

A 41-year-old man had a tolerable pain in his scapular region over a 10 years' period. The pain developed gradually with shoulder motion, in particular with golf swing since he was aiming a professional golf player career. On physical examination, "clunking" was noted once from 90 degrees of abduction to 180 degrees of shoulder motion. A trans-scapular roentgenogram and computed tomography images revealed an osteochondroma located at the anterior and inferior aspect of the scapula. Removal of the tumor was performed by the use of endoscopically assisted resection. One portal was made at the lateral border of the scapula to introduce a 2.7-mm-diameter, 30 degrees Hopkins telescope. The tumor was resected in a piece-by-piece manner by the use of graspers through the same portal. Immediately after the operation pain relief was obtained, and the "clunking" disappeared. CT images showed complete tumor resection. The patient could start playing golf one week after the surgery.

**Conclusion:**

Endoscopically assisted resection of osteochondroma of the scapula provides a feasible technique to treat snapping scapula syndrome and obtain early functional recovery with a short hospital stay and cosmetic advantage.

## Background

Osteochondromas are the most common benign bone tumors of the scapula [1,2]. Osteochondromas usually occur on the anterior surface of the scapula and might cause shoulder discomfort such as pain, limited range of motion with/without winging of the scapula [3,4]. Among those symptoms, the snapping scapula syndrome is characterized by painful, audible, and/or palpable abnormal scapulothoracic motion. Since the snapping is caused by the osteochondroma, removal of the tumor is required to regain normal scapulothoracic motion [3].

Regarding removal of the osteochondroma, conventional open excision has slow functional recovery and cosmetic disadvantage due to a large operative incision [5-7]. Recently, endoscopic resection has been introduced as treatment of osteochondroma causing the snapping scapula syndrome. So far only 2 cases with scapular osteochondromas have been reported in the English literature [5,6].

Here, we present a case with a scapular osteochondroma causing snapping scapula syndrome and treated with endoscopically assisted resection, and we discuss the practicality of the technique.

## Case presentation

A 41-year-old man had tolerable pain in his shoulder, which developed gradually over a 10 years' period. The pain occurred with shoulder motion, particularly in relation to his golf swing, since he was aiming at a professional career as a golfer. On physical examination of the scapula, "clunking" was noted once from 90 degrees of abduction to 180 degrees. However, active range of motion (ROM) was normal. There was no winging of the scapula. The upper extremity and shoulder girdle muscle strength was normal.

**Figure 1:**
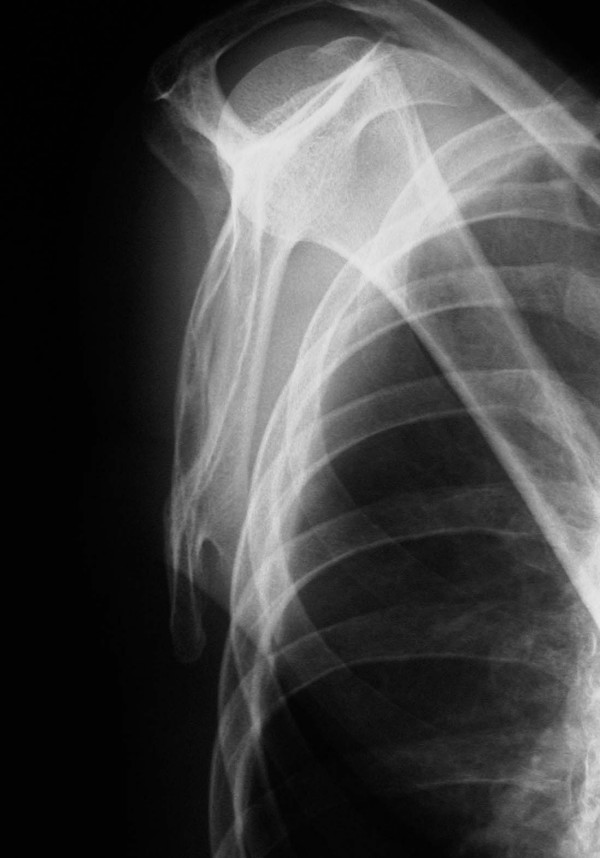
A trans-scapular roentgenogram shows the pedunculated shape of an osteochondorma at the inferior aspect of the anterior scapula.

**Figure 2:**
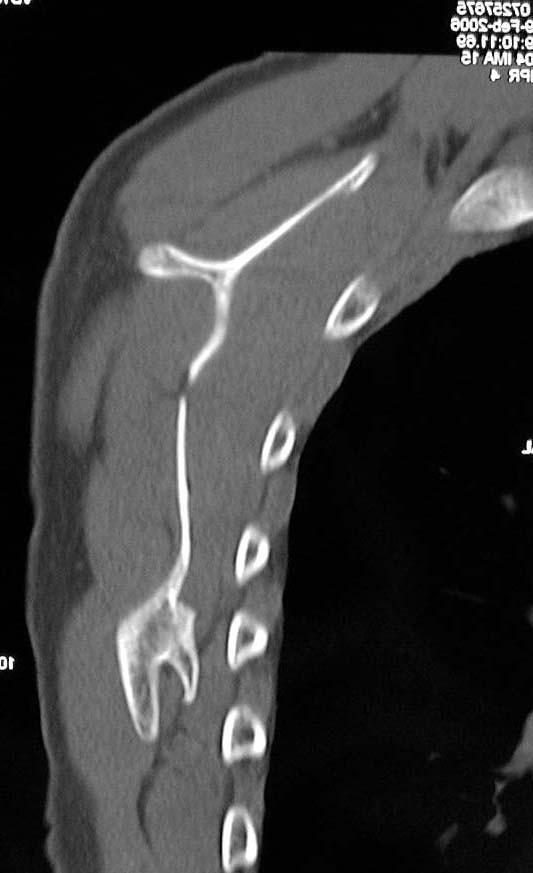
CT image shows an osteochondroma on the anterior and inferior surface of the right scapula.

A trans-scapular roentgenogram showed a pedunculated type of a bony projection, which was continuous with the scapula (Figure 1). Computed tomography (CT) images revealed a bone tumor on the anterior surface of the right scapula and inferior to the spine. The size of the tumor was 1.8 × 1.5 × 1.0 cm. The images demonstrated the continuity between the bone tumor and the scapula (Figure 2). The lesion was diagnosed as an osteochondroma resulting in a snapping scapula syndrome. Removal of the tumor was performed to relieve pain with the "clunking" of the scapula.

### Surgical procedure

**Figure 3:**
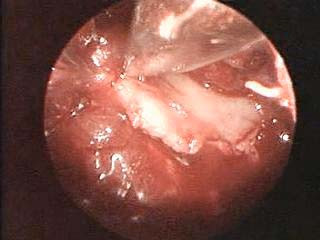
Endoscopic image shows that the tumor was removed by use of a grasper.

Under general anesthesia, the patient was placed in a prone position, with the right arm draped free to allow a full range of motion. The lesion was approached through a 2-cm incision along the lower lateral aspect of the scapula. The teres major muscle was dissected on the same line. Then scapula was elevated by a retractor and then the 2.7-mm-diameter, 30 degrees Hopkins telescope (Karl Storz, Tuttlingen, Germany) was introduced into this portal. Since the tumor was covered by the subscapularis muscle, a part of the muscle was removed to access the tumor by use of graspers. Consequently, the tumor was visualized and resected in a piece-by-piece manner by the use of graspers of different sizes (Figure 3). Finally, a Black Max cutting burr (Anspach, Florida, USA) was applied to smooth the remnants of the tumor. Both the graspers and the burr were introduced through the same portal. The wound was closed over a suction drain, which was removed after 24 hours. The duration of surgery was 2 hour and a half. The patient did not need shoulder immobilization with sling.

**Figure 4:**
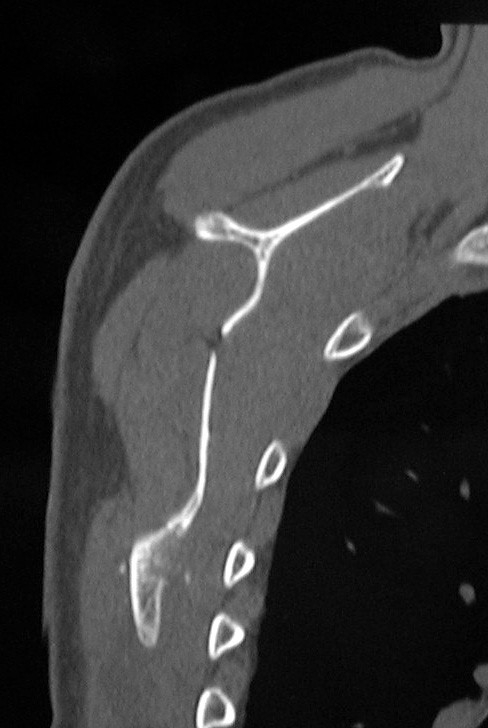
Postoperatively, CT reveals the complete resection the tumor.

**Figure 5:**
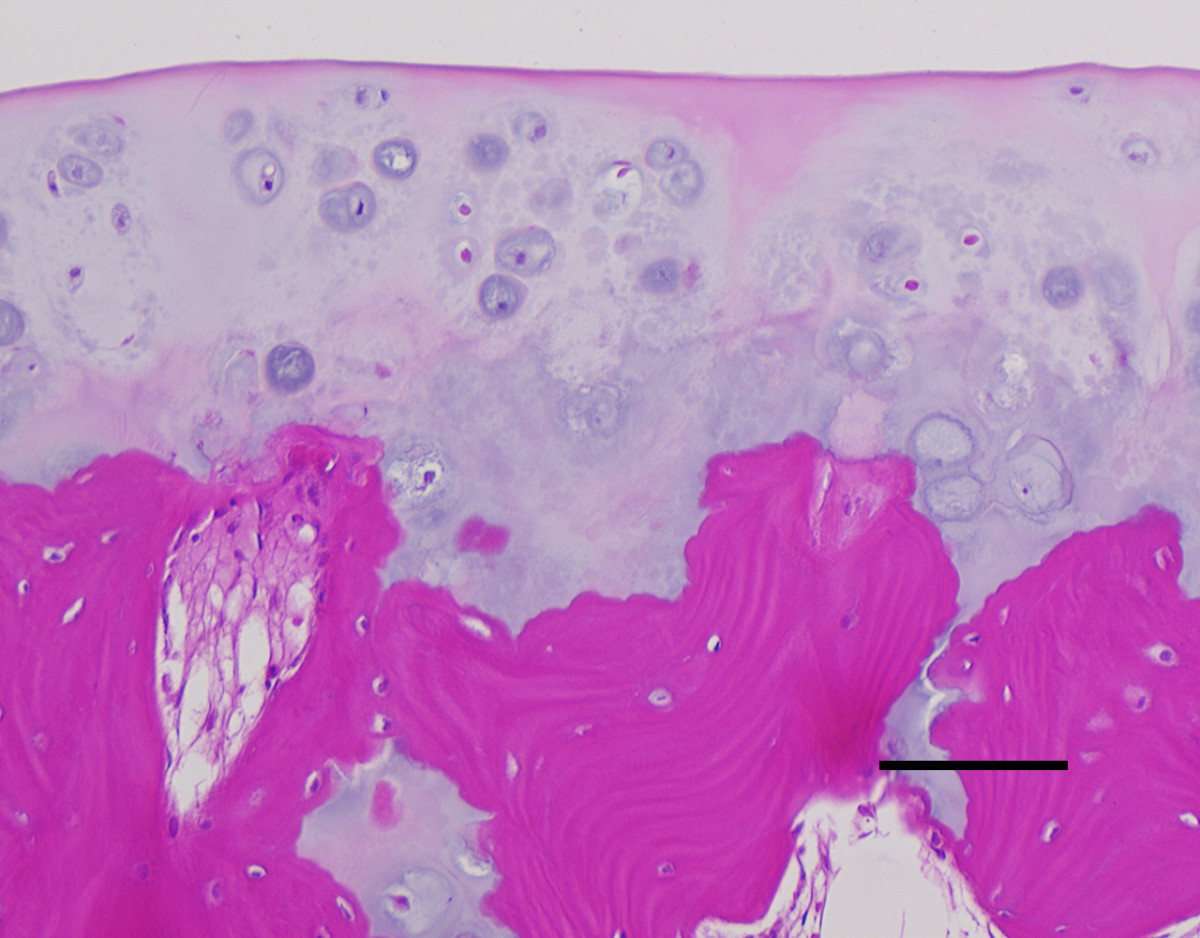
Histology reveals a regular cartilage cap without malignant transformation. (Straight line: 100 μm).

**Figure 6:**
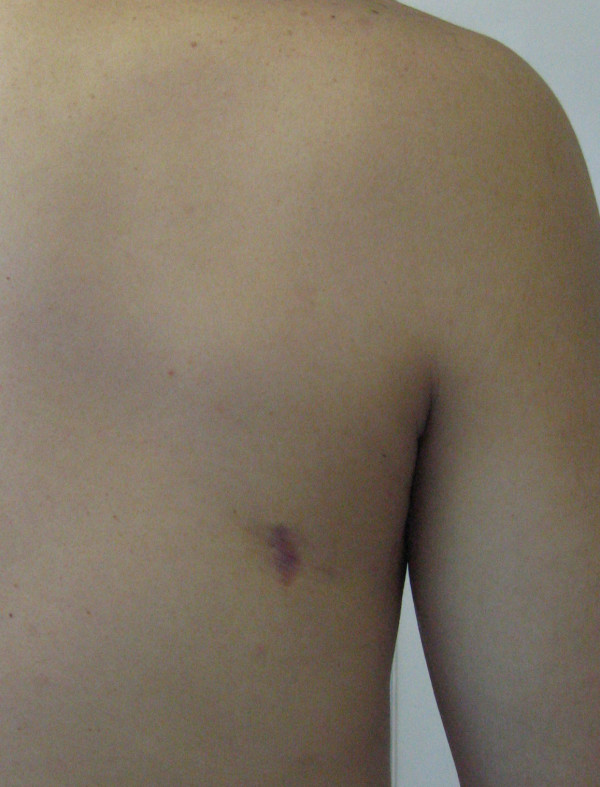
The small scar, 2 cm in length, can be seen in the lateral aspect of the right scapula.

Immediately after the operation pain relief was obtained, and the "clunking" disappeared during full range of active shoulder motion. CT images showed complete tumor resection (Figure 4). The patient could start playing golf one week after the surgery. Histology confirmed the clinical diagnosis of osteochondroma (Figure 5). The scar was small to which the patient was satisfied (Figure 6).

## Discussion

Osteochondromas frequently occur in the metaphyseal region of the long bones. In a series from the Mayo clinic of 872 cases, approximately 86% of the cases (n = 747) had solitary lesions. The scapula was involved in 33 cases, approximately 3.8% of the Mayo clinic series [8]. Among the scapular bone tumors, the osteochondromas are the most common ones [1,2]. With regard to clinical manifestations, many of the osteochondromas are asymptomatic. However, the snapping scapula syndrome might develop when an osteochondroma occurs on the anterior surface of the scapula.

A scapular osteochondroma might cause the snapping scapula syndrome in adolescence or early adulthood [9]. In the present case, however, the clinical manifestation occurred in the fourth decade of the patient's life. We believe that the symptom might have developed in relation to enforcement of the scapular muscle by training. With this regard, Milch [3] mentioned that the strong scapular muscles enforce the contact between the scapular osteochondroma and the ribs, which might worsen the problem.

The snapping scapula syndrome was first described by Boinet in 1867. The subject was fully reviewed by Milch and Burman in 1933 [10]. The causes of the syndrome have been classified as abnormalities of the bone, muscle, or bursa which are involved in the scapulothoracic movement [3]. Carlson *et al*., [11] reviewed 89 cases in English literatures between 1867 and 1996. Osteochondroma was a leading cause among bone abnormalities, accounting for 14 out of the 89 cases (15.7%).

Both conservative and surgical approaches have been applied to treat this condition. The conservative treatment including immobilization of the scapula, physiotherapy, or local anesthetic injection might be effective in cases where only the soft tissues are involved. In contrast, when the cause of the snapping scapular syndrome is a bony abnormality, the involved area must be resected. Recently, endoscopic resection has been introduced as treatment of painful snapping scapula caused by bone abnormalities [5,6].

Historically, the extra-articular endoscopic resection of the bone tumors was first introduced and applied in the case of a chondroblastoma of the femoral head in 1995 [12,13]. Of osteochondromas, only one in the distal femur [7] and only 2 in the scapula have been reported [5,6]. The authors emphasized the superiority of endoscopically assisted tumor resection to the traditional surgical treatment [5-7]. By the use of endoscopy, the size of the incision, the amount of sacrificed soft tissue, and the blind areas can be efficiently reduced. Thus, the entire tumor resection is achieved even through a small incision. Consequently, the patient can obtain an early functional recovery and a cosmetic advantage.

In order to safely achieve complete tumor resection through a small incision, the most important factors are the location and the number of the portals. Ruland *et al*., [14] described the relation of major neurovascular structures to safe portal sites based upon a cadaveric study. The authors recommended that the portals should be inferior to the spine of the scapula to avoid the neurovascular structure at the superomedial angle. In addition, an area of three to four fingerbreadths from the medial border of the scapula is required to avoid the dorsoscapular nerve and artery. Harper *et al*., [6] applied the method of Ruland [14] to treat one case of an osteochondroma located at the superomedial angle. On the other hand, in the case of Kumar [5], the portal was made at the lateral aspect of the scapula and through the axillar region to treat the osteochondroma at the superomedial angle. A portal by the lateral approach has the advantage of not being in close proximity to major nerovascular structures. In addition, only one muscle, the teres major, needs to be partially sectioned. In the present case, the tumor was located inferior to the spine of the scapula. Only one lateral portal was needed to observe and remove the entire tumor. The other 2 cases reported were also treated by one portal. However, more than 2 portals should be made when the tumor is not well visualized or difficult to remove.

## Conclusion

A case with snapping scapula syndrome due to an osteochondroma was successfully treated with an endoscopically assisted resection by the use of one lateral portal. This is a feasible technique to obtain early functional recovery with short hospital stay and cosmetic advantage.

## Competing interests

The author(s) declare that they have no competing interests.

## Authors' contributions

**SF **collected the data and has written up the manuscript, **HF **had the idea and granted permission to use his patient data for preparing the manuscript, and finalized writing of the manuscript. **SY **helped in scrutiny of the paper.

All authors read and approved the final manuscript.
